# GATA4 promotes hepatoblastoma cell proliferation by altering expression of miR125b and DKK3

**DOI:** 10.18632/oncotarget.12839

**Published:** 2016-10-24

**Authors:** Yihua Pei, Qin Yao, Sibo Yuan, Bozhen Xie, Yan Liu, Chunsheng Ye, Huiqin Zhuo

**Affiliations:** ^1^ Central Laboratory, The Affiliated Zhongshan Hospital, Xiamen University, Xiamen, Fujian 361004, China; ^2^ Department of Gastrointestinal Surgery, The Affiliated Zhongshan Hospital, Xiamen University, Xiamen, Fujian 361004, China; ^3^ Department of Spine Surgery, The Affiliated Zhongshan Hospital, Xiamen University, Xiamen, Fujian 361004, China; ^4^ Department of Pathology, The Affiliated Zhongshan Hospital, Xiamen University, Xiamen, Fujian 361004, China; ^5^ Department Otolaryngology, The Affiliated Zhongshan Hospital, Xiamen University, Xiamen, Fujian 361004, China

**Keywords:** hepatoblastoma, GATA4, miR125b, DKK3

## Abstract

GATA4 is a zinc finger DNA-binding protein that plays an important role in mammalian liver development. However, the effects of GATA4 in hepatoblastoma (HB), a common liver cancer in pediatric patients, remain largely unknown. In this study, we demonstrate that GATA4 promotes growth and survival in the Huh6 human hepatoblastoma cell line. GATA4 expression was high in Huh6 cells, and its knockdown decreased expression of Dickkopf-related protein 3 (DKK3), a gene that may contribute to premature or undifferentiated phenotypes in HB. GATA4 also directly bound to the promoter regions of the miRNA miR125b and inhibited its expression in Huh6 cells. DKK3 was a direct target of miR125b in Huh6 cells. Inhibition of miR125b or overexpression of DKK3 promoted proliferation, survival, migration, and invasion in Huh6 cells. This is the first report to demonstrate that GATA4 promotes oncogenesis by inhibiting miR125b-dependent suppression of DKK3 expression. This GATA4/miR125b/DKK3 axis may be a major regulator of growth, migration, invasion, and survival in hepatoma cells, and is therefore a potential therapeutic target or biomarker for progression in HB patients.

## INTRODUCTION

Hepatoblastoma (HB) is the most common pediatric liver tumor in children under 5 years old [4] and accounts for approximately 1-4% of all malignant tumors in children [1, 2]. 0.5-1.5 children per million are diagnosed with HB each year [3], and incidences have increased in recent years. Many genetically inherited syndromes, including glycogen storage disease, familial adenomatous polyposis, and Beckwith-Wiedemann Syndrome, are important risk factors for HB. During HB development, malignant tumor cells arise from differentiated and proliferating pluripotent stem cells that are important for embryonic development [5, 6]. The molecular mechanisms underlying HB tumorigenesis are more complex. The overexpression or mutation of several developmental signaling molecules, and alterations in the activity of related pathways, contributes to HB pathogenesis. For example, genetic aberrations in the Wnt signaling pathway are found in a majority of HB cases. Mutations in the β-catenin gene (*CTNNB1*) [7] and the resulting uncontrolled activation of Wnt signal target gene (Myc, survivin) expression, as well as mutations in telomerase reverse-transcriptase (TERT) promoter regions, promote the development of, and aggressive phenotypes in, HB [8]. In general, oncogenic signaling pathways activate the expression of a set of transcription factors (TFs). This deregulation of TFs contributes to tumor development by promoting the expression of oncogenic proteins and inhibiting tumor suppressor genes [9]. Many TFs, including STAT family members, serum response factors, NF-kB, and AP1, contribute to the development of various human cancers in this way, while the expression of many tumor suppressor proteins, such as GATA4 and Dickkopf-related proteins, is inhibited during carcinogenesis [10-12]. Despite recent progress in identifying the cellular events and mechanisms underlying the development of various liver cancers, the molecular mechanisms of HB development and progression have not been fully explored.

GATA family transcription factors are zinc finger DNA-binding proteins which play a vital role in the regulation of cell growth and differentiation. GATA4, 5, and 6 are predominantly expressed in organs originating from mesoderm or endoderm, such as the liver, lungs, heart, and gut [13], and GATA4 specifically contributes to mammalian liver development [14]. In humans, GATA4 is expressed in hepatocytes during early gestation, but is subsequently expressed only in endothelial cells surrounding the hepatic vessels and in Kupffer cells [15]. Loss of GATA4 expression occurs in many cancers, including epithelial ovarian cancer, adrenocortical tumors, hepatocellular carcinoma, and colorectal cancer, indicating that GATA4 may be a tumor suppressor [16]. In contrast, GATA4 expression is elevated in the Huh6 pediatric liver tumor cell line [17]. However, the effects of GATA4 expression on HB development and progression are not well-understood [17]. Dickkopf-related protein-3 (DKK3) is one of the proteins expressed during the very early stages of hepatogenesis. DKK3 suppresses Wnt-dependent carcinogenic activity by reducing nuclear and cytoplasmic β-catenin accumulation [18]. However, hypermethylation of the DKK3 promoter region suppresses its expression in many cancer cells, including hepatocarinoma [19, 20].

Some reports suggest that DKK3 is a divergent member of the Dkk family because its function is poorly understood and it has little effect on Wnt/β-catenin signaling. In contrast, DKK3 has also been identified as a potential marker for angiogenesis. DKK3 protein is highly expressed in the blood vessels of gliomas, non-Hodgkin lymphomas, colorectal cancer, and melanoma, but not in matched normal tissues [21, 22]. Additionally, DKK3 overexpression may be related to the premature development or undifferentiated nature of HB in some patients [23]. Due to its abnormal expression pattern, whether DKK3 acts as an oncogenic gene in HB patients remains unclear; further study is required to elucidate the function of DKK3, and its potential as a tumor therapy, in HB.

microRNAs (miRNAs), a class of small non-coding RNAs, are implicated in carcinogenesis and tumor progression in various types of cancer, including liver cancer [24]. The expression patterns of many miRNAs are altered in hepatoblastoma patients, and this alteration plays a crucial role in HB pathogenesis [25, 26]. The miRNA-125 family (miRNA-125a-3p, miRNA-125a-5p, miRNA-125b-1, and miRNA-125b-2) is highly-conserved in many species, and expression of these miRNAs regulates carcinogenesis and tumor development by targeting many tumor-promoting and -suppressing transcription factors [27, 28]. Specifically, the inhibitory effects of miR125b on human cancer cell proliferation and metastasis have been extensively investigated [29]. miR125b is down-regulated in liver cancer, and overexpression of miR125b inhibits cell cycle progression and induces cell death in HCC by targeting Mcl-1 and IL-6R. In addition, miR125b regulates invasion and migration in HCC [30-32]. Several oncogenic molecules, including LIN28B, placenta growth factor (PIGF), and SIRT7, are downstream targets of miR125b [33-35]. Despite its tumor suppressor role in liver cancer, the mechanism by which miR125b is downregulated in hepatocarcinoma, especially in HB, remains unknown.

Here, we demonstrated that GATA4 upregulation in Huh6 HB cells was associated with increases in proliferation, invasion, and metastasis both *in vitro* and *in vivo*. Moreover, our results suggested that GATA4 promotes Huh6 progression by suppressing miR125b transactivation. Furthermore, we identified DKK3 as a direct target of miR125b. Although GATA4 and DKK3 acted as tumor suppressors in most carcinomas, they promoted tumor progression in HB. Importantly, we found that GATA4 acted as an upstream factor to inhibit miR125b and DKK3 activation during HB cell proliferation. These results not only provided evidence for a novel mechanism that regulated HB progression, but also suggested that the aggressive nature of HB was due, at least in part, to interactions between GATA4, miR125b, and the oncogenic gene DKK3.

## RESULTS

### DKK3 promotes migration and invasion in Huh6 cells

**Figure 1:**
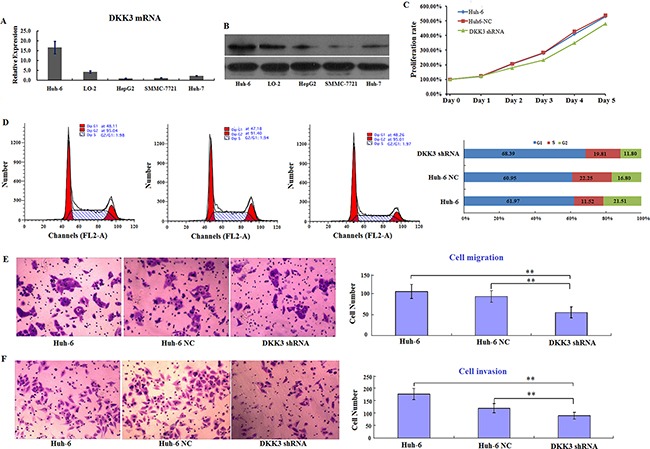
DKK3 promotes proliferation and survival in Huh6 cells *in vitro* **A.** Relative levels of DKK3 mRNA expression in five different hepatic cell lines. Expression is normalized to the 18s rRNA expression value. The values are expressed as mean ± standard deviation of three samples. **B.** Western blot analysis of DKK3 expression in five different hepatic cell lines (left) and DKK3 and GAPDH (control) protein levels in Huh6 cells, Huh6 NC cells, and DKK3 shRNA-transfected cells (right). **C.** Proliferation curves for Huh6 cells 5 days after transfection with NC or DKK3 shRNA. **D.** Cell cycle analysis for Huh6 cells after transfection with NC or DKK3 shRNA (left) and the distribution of cells in different phases (right). **E.** Transwell migration assay using Huh6 cells transfected with NC or DKK3 shRNA (left); numbers of migrated cells in each group are shown (right). **F.** Transwell invasion assay using Huh6 cells transfected with NC or DKK3 shRNA (left); numbers of invaded cells in each group are shown (right). Error bars represent standard deviations for three repeated samples. Huh6 NC: Huh6 cells treated with negative control shRNA; DKK3 shRNA: Huh6 cells treated with DKK3 shRNA. **P<0.01.

Although DKK3 expression is down-regulated in most cancers, a recent study demonstrated that DKK3 is overexpressed in some HB patients, which is inconsistent with its typical tumor suppressor role [23]. However, it is unclear whether the upregulation of DKK3 is a cause of or a response to oncogenesis in HB. Here, we examined DKK3 expression patterns in the following cell lines: normal liver hepatocyte LO-2 cells, Huh6 cells (HB cell line), and human hepatocarcinoma cell lines (HepG2, SMMC-7721, and HuH7). Similar to other cancers, DKK3 expression was lower in the hepatocellular carcinoma cell lines (HepG2, SMMC-7721, and HuH7) than in normal liver cells (LO-2). Interestingly, DKK3 expression was much higher in HB cells (Huh6) than in normal liver cells (Figure 1A and 1B), which agreed with previous observations in HB patient liver samples [23] and indicated that DKK3 may play a unique role in HB. Next, we examined the effects of DKK3 on carcinogenic activity in HB by inhibiting its expression in Huh6 cells with a lentiviral DKK3 shRNA vector. This vector was effective in decreasing DKK3 expression (Figure 1B); empty vector-treated cells were used as a negative control (NC). Proliferation rate also decreased in DKK3 shRNA-transfected Huh6 cells (Figure 1C), as did the number of cells in the G2 phase of the cell cycle (Figure 1D). Next, we examined the influence of DKK3 on migration and invasion in HB cells. DKK3 knockdown markedly decreased migration in Huh6 cells compared to non-transfected cells in a transwell migration assay (Figure 1E). Similarly, shRNA-mediated inhibition of DKK3 reduced invasion in Huh6 cells in a transwell invasion assay (Figure 1F). These results suggest that DKK3 plays an important role in the development of HB.

**Figure 2:**
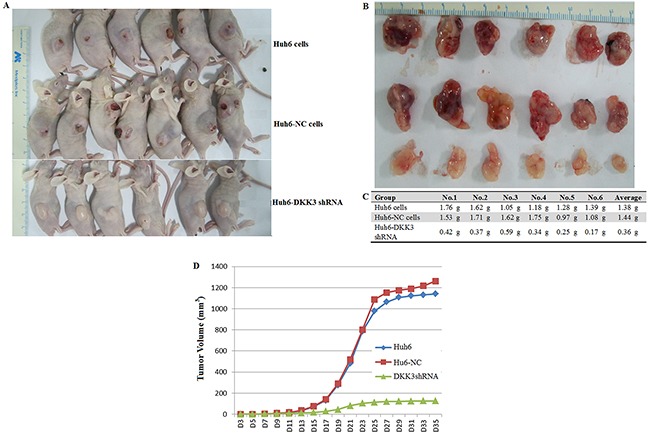
DKK3 knockdown inhibits tumorigenesis *in vivo* **A.** Images of sacrificed nude mice bearing tumors 35 days after receiving transplants of Huh6 cells transfected with NC or DKK3 shRNA. **B.** Images of tumors from the sacrificed mice. **C.** Tumor weights. **D.** Tumor volume growth curves for nude mice 35 days after receiving Huh6 cell transplants.

To confirm that DKK3 also plays a tumorigenic role *in vivo*, Huh6 cells transfected with DKK3 shRNA or empty viral vector were transplanted into nude mice. Tumors were collected 35 days after transplantation (Figure 2A and 2B). Tumor sizes, volumes, and weights were lower in DKK3 shRNA group mice than in mice from either control group (negative control and non-transfected) (Figure 2B-2D). These results indicate that DKK3 is required for HB tumor proliferation *in vivo*. Together, these *in vitro* and *in vivo* data suggest that DKK3 promotes proliferation, migration, and survival in hepatoblastoma cells. In addition, our data reveal that inhibition of DKK3 inhibits HB progression and invasion.

### miR125b suppresses proliferation, migration, and invasion in Huh6 cells

**Figure 3:**
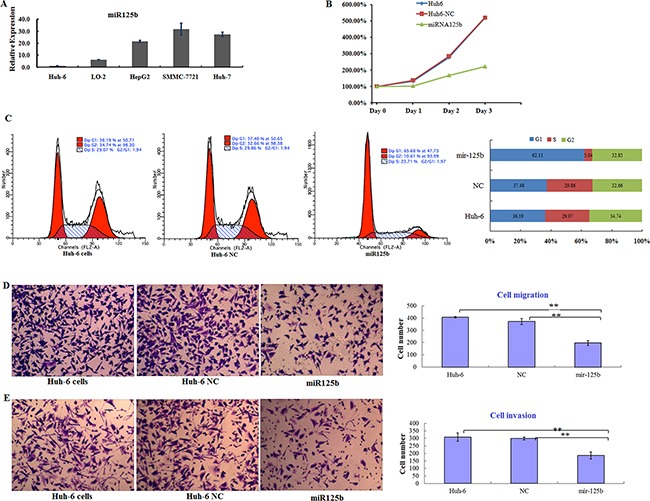
miR125b is downregulated in HB cells and inhibits cell proliferation and survival *in vitro* **A.** Relative miR125b expression in five cell lines normalized to U6 expression. Error bars represent standard deviations for three repeated samples. **B.** Proliferation curves for Huh6 cells 3 days after transfection of control miRNA (NC) or miR125b. **C.** Cell cycle analysis for Huh6 cells after transfection of NC or miR125b (left) and the distribution of cells in different phases (right). **D.** Transwell migration assay using Huh6 cells transfected with NC or miR125b (left); numbers of migrated cells in each group are shown (right). **E.** Transwell invasion assay using Huh6 cells transfected with NC or miR125b (left); numbers of invaded cells in each group are shown (right). Error bars represent standard deviations for three repeated samples.

miR125b is an important regulator of oncogenic protein expression, and experimental evidence increasingly indicates that it might be a useful diagnostic biomarker of, and therapeutic target in, several types of cancer [36]. Aberrant miR125b expression induces cell proliferation and blocks apoptosis in many cancers. In contrast, miR125b acts as a tumor suppressor in breast cancer and HCC, in which its expression is down-regulated [37]. However, the role of miR125b in HB is still unclear. Therefore, we examined miR125b expression and function in Huh6 cells. RT-PCR revealed that miR125b expression was decreased by more than 90% in Huh6 cells, but was increased in hepatocarcinoma cell lines (HepG2, SMMC-7721, and HuH7) (Figure 3A). This miR125b expression pattern was the opposite of the DKK3 expression pattern observed in the cell lines examined. Next, we examined the function of miR125b in Huh6 cells. Ectopic miR125b expression reduced cell proliferation rates compared to control cells (Figure 3B). In miR125-expressing Huh6 cells, cell cycle progression at the G0/G1 phase decreased approximately 2-fold (Figure 3C). Cell migration (Figure 3D) and invasion (Figure 3E) in also decreased in miR125b-expressing Huh6 cells compared to control cells in transwell assays. Taken together, these findings suggest that miR125b suppresses progression in HB cells by inhibiting proliferation and survival. In addition, our results indicate that miR125b and DKK3 exert opposite effects in HB cells.

### DKK3 is a downstream target of miR125b

**Figure 4:**
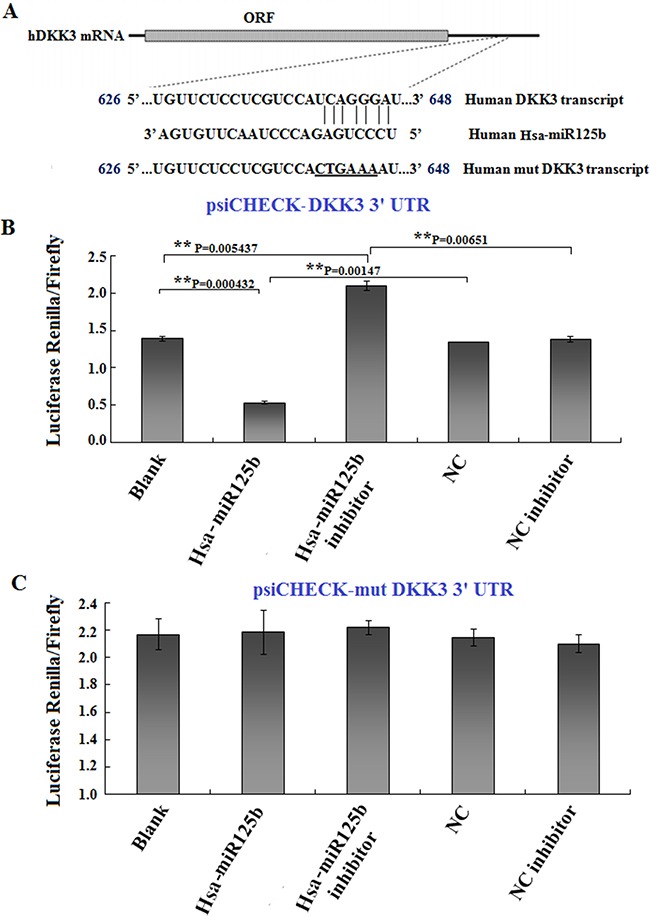
DKK3 is a target of miR125b **A.** Illustration of the predicted target sequence of miR125b located in the 3′-UTR of DKK3 mRNA. “UCAGGGA” in the DKK3 transcript represents the seed sequence, which was mutated to “CTGAAA” to construct the mutant DKK3 transcript. **B, C.** Luciferase constructs (0.5 μg) with wild-type (B) or mutated (C) DKK3 3′UTRs were transfected into 293T cells, and luciferase activity was measured 24 hr after transfection. Blank: 293T cells; Hsa-miR125b: 293T cells treated with 50 nM miR125b; Hsa-miR125b+inhibitor: 293T cells treated with 50 nM miR125b and 100 nM miR125b inhibitor; NC: 293T cells treated with 50 nM scrambled miRNA; NC inhibitor: 293T cells treated with 100 nM scrambled miRNA inhibitor. Luciferase values are normalized to the NC group. Average activity from five repeated samples were used to calculate inhibition percentages. Error bars represent the standard errors of the mean for five independent experiments.

Recent studies indicated that miRNAs regulate DKK3 expression. Because we found that miR125b and DKK3 had opposite expression patterns and functions in Huh6 cells, we hypothesized that DKK3 was a target of miR125b. To explore this possibility, we performed an *in silico* analysis to identify miRNAs that are predicted to target the 3′UTR of the DKK3 transcript, which is approximately 1000 bp in length. Several online software programs, including PicTar, TargetScan, and Microna, predicted that the sequence between nucleotides 626 to 648 is likely targeted by miRNA125b (Figure 4A). To determine whether miR125b targeted the predicted DKK3 3′UTR sequence, a luciferase reporter containing the wild-type DKK3 3′UTR was constructed. Using this construct as a backbone, the UCAGGG nucleotides (Figure 4A) in the seed region of the predicted binding site were mutated to CTGAAA (underlined sequence in Figure 4A). The wild-type and mutant luciferase reporters were transfected into 293T cells along with Hsa-miR125b, Hsa-miR125b inhibitor, or both. Luciferase activity was measured 48 h after transfection. As shown in Figure 4B, miR125b reduced wild-type DKK3-3′UTR luciferase activity, and this inhibition was reversed in the presence of miR125b inhibitor. In contrast, miR125b did not affect luciferase activity in cells with mutations in the DKK3-3′UTR seed region (Figure 4C). These results suggest that miR125b downregulates DKK3 expression by directly binding to the nucleotide sequence between 626 and 648 in the 3′UTR region of DKK3 mRNA.

### GATA4 inhibits miR125b transcription by directly targeting the miR125b promoter region

**Figure 5:**
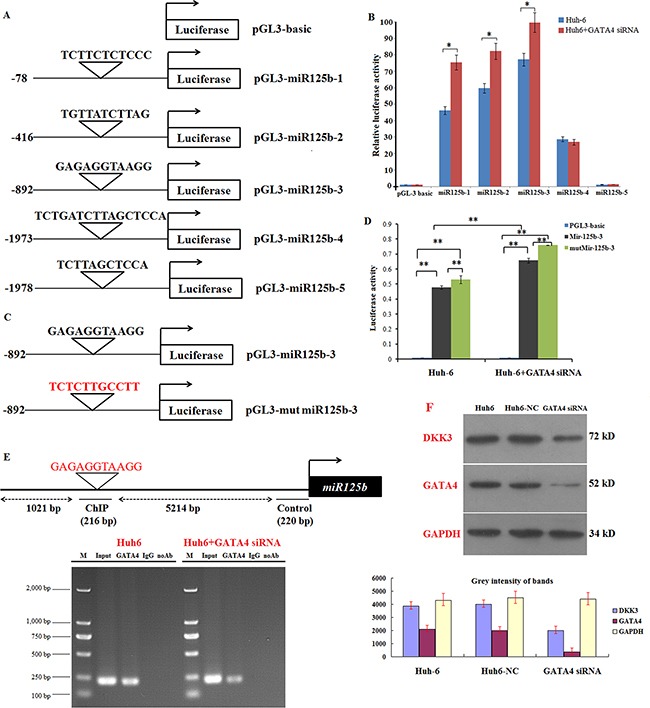
GATA4 trans-inactivates miR125b expression and indirectly regulates DKK3 expression **A.** Schematic illustration of 5 putative wild-type miR125b promoter constructs fused to a luciferase reporter gene using pGL-3 plasmid vector. **B.** Luciferase activity in extracts in Huh6 cells treated with or without GATA4 siRNA (Huh6 GATA4 siRNA) and transiently transfected with the 5 wild-type miR125b luciferase reporter constructs. Luciferase values are normalized to the Huh6 cells transfected with the empty pGL-3 basic vector. **C.** Schematic illustration of human wild-type or mutant miR125b-3 promotor constructs fused to a luciferase reporter gene. **D.** Luciferase activity in extracts from Huh6 cells treated with or without GATA4 shRNA cells and transiently transfected with the wild-type or mutant miR125b-3 luciferase reporter constructs. Average activity from five repeated samples was used to calculate luciferase activity. **E.** Schematic of the miR125b-3 upstream promoter containing a single GATA4-binding site. ChIP analyses revealed that GATA4 binds to the upstream GATA boxes in Huh6 cells; binding activity decreased in Huh6 cells treated with GATA4 siRNA. **F.** Western blot analysis of DKK3 and GATA4 expression in Huh6 cells treated with or without GATA4 siRNA cells; GAPDH served as the control. Huh6 NC: Huh6 cells treated with negative control miRNA. *P<0.05; **P<0.01.

GATA4 target genes are characterized by the presence of the GATA4-binding consensus element, which is called the GATA box. Recent studies estimate that more than one-fourth of mammalian miRNA genes contain at least one GATA box in their promoter region. To examine whether miR125b is a target of GATA4 during HB development, we analyzed the miR125b promoter sequence to identify possible binding sites for GATA4. Five putative GATA4 binding sites in miR125b were identified using the JASPAR dataset with a high score (85%) setting (Figure 5A). Based on this prediction, we constructed 5 luciferase reporter plasmids containing wild-type putative GATA4-binding sites upstream of the miR125b coding sequence (pGL3-miR125b-1, pGL3-miR125b-2, pGL3-miR125b-3, pGL3-miR125b-4 and pGL3-miR125b-5). These constructs were transfected into Huh6 cells to determine whether miR125b transcription is inactivated by GATA4 in HB cells. Luciferase activity was higher in Huh6 cells transfected with the pGL-miR125b-3 promoter (starting from -892) compared to the other constructs (Figure 5B). Notably, siRNA-mediated GATA4 knockdown increased luciferase activity after transfection with all miR125b promoter constructs except promoter pGL3-miR125b-5. To confirm the interaction between GATA4 and the miR125b promoter, we next transfected Huh6 cells with plasmids in which the miR125b-3 promoter seed region nucleotides were mutated from GAGAGGTAAGG to TCTCTTGCCTT (red sequences in Figure 5C). Luciferase activity, which increased after transfection with the wild-type miR125b-3 promoter, was further enhanced in cells transfected with the mutant miR125-3 promoter. In addition, siRNA-mediated GATA4 knockdown increased luciferase activity after transfection with both wild-type and mutant- miR125b-3. These results confirmed that GATA4 interacts with miR125b (Figure 5D). Chromatin immunoprecipitation (ChIP) analysis revealed that GATA4 specifically bound to the GATA element in the miR125b promoters, and GATA4 knockdown markedly reduced binding in Huh6 cells (Figure 5E). GATA4 knockdown also reduced DKK3 expression in Huh6 cells, indicating that GATA4 promotes DKK3 expression by suppressing miR125b. We next examined whether GATA4 affects the expression of DKK3. GATA4 expression is high in Huh6 cells (Supplementary Figure S1), and GATA4 knockdown reduced Huh6 cell proliferation, invasion, and migration (Supplementary Figure S2). GATA4 overexpression decreased miR125b mRNA levels in Huh6 cells (Supplementary Figure S3A), and GATA4 knockdown in cells overexpressing miR125b dramatically reduced DKK3 mRNA and protein levels (Supplementary Figure S3A and S3B). Collectively, these results provide strong evidence that GATA4 directly binds to the miR125b promoter region and inhibits its expression. Thus, GATA4 indirectly activates oncogenic DKK3 activity by inhibiting miR125b expression.

## DISCUSSION

Although HB and HCC are the most common malignant pediatric liver tumors and are characterized by aggressive growth and early metastasis, the molecular mechanisms underlying their development remain largely unknown [38]. Numerous oncogenic molecules and signaling pathways are co-regulated by functionally-related TFs and associated genes [39]. In this study, we investigated the functions of GATA4 and its oncogenic pathway in the Huh6 HB cell line. GATA4 promotes Huh6 cell proliferation by blocking the transcription of miR125b, which in turn increases DKK3 expression. This is the first study to report the interaction among these three molecules in carcinoma and reveals a novel connection between miRNAs and transcription factors in HB development and proliferation. Thus, the GATA4/miR125b/DKK3 axis might serve as a novel therapeutic target in pediatric hepatoblastoma patients.

GATA4 is abundantly expressed in various cancers and plays a crucial role in their development. For example, GATA4 enhances survival in liver cancer cells [40]. Here, we found that GATA4 expression was increased in the Huh6 human HB cell line, but not in other HCC cell lines. This might be due to the similarity of HB tissue to typical fetal liver tissue, which also has high GATA-4 expression, especially during the early gestational stage [15]. Accordingly, Makkru *et al*. reported that GATA4 expression and activity were high in liver tumors in children, but not in adults. Furthermore, transcription factors and miRNAs are interconnected in gene regulatory networks in multicellular organisms [41], and the promoter regions of most mammalian miRNA genes contain at least one GATA box, indicating that some miRNAs may be targets of GATA4 [42]. Here, we found a negative association between GATA4 and miR125b expression in Huh6 cells. Furthermore, we confirmed that GATA4 binds to the miR125b promoter, thereby suppressing miR125b transcription and expression in Huh6 cells. This is the first evidence of this interaction between GATA4 and miR125b.

miR125b, which has various functions and acts as a tumor suppressor, has been examined in many tumors. Zhao *et al*. found that miR125b inhibited proliferation and cell cycle progression in hepatocellular carcinoma cells by targeting Mcl-1 and IL6R [32]. Additionally, Han *et al*. found that miR125b inhibited HCC development by suppressing SIRT7 and cycline D1 expression and inducing p21-dependent G1 cell cycle arrest [33]. Unsupervised hierarchical clustering analysis of 510 of 1,371 miRNAs from eight normal liver tissues and 16 HCCs selected with minimum filtering criteria revealed two distinct clusters in a dendrogram. That study identified miR125b as one of the five most important miRNAs related to HCC progression. Here, *in vitro* assays demonstrated that restoration of miR125b expression suppressed Huh6 cell proliferation, migration, invasion, and metastasis. Moreover, to our knowledge, our study provides the first evidence that miR125b regulates DKK3 expression.

Unlike other members of the DKK family, the receptor for DKK3 has not been identified, and the role of DKK3 in the Wnt/β-catenin pathway remains unclear. Regardless, decreases in DKK3 expression in various cancers suggest that it may act as a tumor suppressor and serve as a therapeutic target [43]. In contrast, we previously found that DKK3 is overexpressed in both HB and HCC, and to a greater degree in the former than in the latter. This suggests that DKK3 might be related to the premature or undifferentiated phenotypes that characterize HB [23]. Furthermore, differences in DKK3 expression may help explain the presence of primarily undifferentiated small hepatic cells in HB and primarily differentiated hepatic cells in HCC. Additionally, HB is mainly a pediatric liver disease, while HCC is primarily an adult liver disease. Although recent studies report that GATA4 is abundantly expressed in pediatric, but not in adult, liver tumors, its exact role in HB remains unknown [17]. Here, we observed a positive association between GATA4 and DKK3 levels in Huh6 cells, and shRNA-mediated GATA4 knockdown decreased DKK3 expression. However, ChIP experiments excluded the possibility that GATA4 promotes DKK3 expression through direct transcriptional mechanisms. Because miRNAs are critical regulators of cell proliferation and survival, we hypothesized that GATA4 might regulate cell growth through the transactivation of miR125b. The following results further confirmed interactions between GATA4, miR125b, DKK3: (1) inhibition of miR125b in GATA4-expressing Huh6 cells reversed GATA4-induced inhibition of DKK3 by GATA4; (2) ectopic miR125b expression suppressed proliferation, decreased DKK3 levels, and reduced proliferation by altering cell cycle progression, apoptosis, and invasion in Huh6 cells. In contrast with the findings of Makkru *et al*., our findings suggest that altering the amount of functional GATA4 in Huh6 cells affects cell proliferation, migration, metastasis, and apoptosis by indirectly regulating of DKK3 expression. Our results thus demonstrate a novel mechanism by which this TF and miRNA co-regulate DKK3 expression and tumor cell growth.

In summary, in this study we investigated the role of GATA4 in HB development and the underlying regulatory mechanisms. Our data suggest that upregulation of GATA4 promotes HB cell survival and growth via the trans-inactivation of miR125b, which leads to DKK3 upregulation and induces proliferation. This GATA4/miR125b/DKK3 pathway represents a novel mechanism that drives HB development and might serve as an effective therapeutic target for HB treatment.

## MATERIALS AND METHODS

### Cell lines and cell culture

The LO6 cell line was obtained from the American Type Culture Collection; Huh6, SMMC7721, HepG2, and Huh7 cell lines were provided by the China Center for Type Culture Collection. Huh6, HepG2, and Huh7 cells were maintained in DMEM supplemented with 10% fetal bovine serum (GIBCO, NY, USA); LO6 and SMMC7721 cells were maintained in RPMI 1640 supplemented with 10% fetal bovine serum and 1% ampicillin/streptomycin.

### RNA extraction and real-time RT-PCR

Total cellular RNA was extracted using TRIzol reagent (Life Technologies, Carlsbad, CA, USA). The RT-PCR primers for miR125b and U6 snRNA were purchased from RiboBio (Guangzhou, China). Reverse transcription PCR was performed using the PrimeScript RT Reagent Kit (TaKaRa, Dalian, China) according to the manufacturer's instructions. The PCR primers were as follows: DKK3 forward, 5′- ACACAGACACGAAGGTTGGA-3′; DKK3 reverse, 5′- CGTCTCCCACAGATGTGATA-3′; 18s rRNA forward, 5′- CCTGGATACCGCAGCTAGGA-3′, 18s rRNA reverse, 5′-GCGGCGCAATACGAATGCCCC-3′; hsa-miR125b forward, 5′-ACACTCCAGCTGGGTCCCTGAGACCCTAACTTTCCCTGAG-3′, hsa-miR125b reverse, 5′- CTCAACTGGTGTCGTGGA-3′; U6 forward, 5′-CTCGCTTCGGCAGCACA-3′, and U6 reverse, 5′- AACGCTTCACGAATTTGCGT-3′.

PCR consisted of an initial denaturing step at 95°C for 1 min, followed by 40 cycles of 95°C for 30 sec, 60°C for 1 min, and 72°C for 1 min, and a final extension step at 72°C for 7 min. Real-time PCR was performed using SYBR Premix Ex Taq II (TaKaRa) and a Light Cycler 480 system (Roche, Basel, Switzerland). U6 level was used as an internal control. The 2^−ΔΔCT^ method was used to determine fold changes in RNA levels in each sample compared to the reference sample.

### Small interfering RNA (siRNA) transient transfection

The siRNA oligonucleotide sequences targeting Dkk3 and GATA4 were purchased from Riobio (Guangzhou, China). 100 nM of Dkk3 siRNA or GATA4 siRNA was used to knock down the expression of Dkk3 or GATA4 using Oligofectamin reagent (Invitrogen) according to the manufacturer's instructions. Scrambled siRNA was used as the control. Silencing of Dkk3 or GATA4 expression was confirmed by western blot analysis with anti-Dkk3 or -GATA4 antibodies.

### Plasmid and oligonucleotide transfection

The pcDNA3-DKK3 plasmid was constructed as previously described by inserting DKK3 cDNA into the pcDNA3 vector (Life Technologies) at the Xhol and Xbal sites. Cells were plated in 60 mm dishes and allowed to reach 60% confluence prior to transfection.

To construct promoter reporter vectors, the wild-type miR125b promoter containing the putative binding site for DKK3 was PCR-amplified using genomic DNA from Huh6 cells as a template. The DKK3 binding sites in miR125b were subjected to site-directed mutagenesis to create mutant constructs. Both wild-type and mutant promoters were inserted upstream of the Firefly luciferase reporter in the pGL3-basic vector. To construct miRNA 3′-UTR luciferase reporter vectors, the wild-type 3′-UTR of GATA4, containing putative binding sites for miR125b, was PCR-amplified using genomic DNA from Huh6 cells as a template. The corresponding mutant constructs were created by mutating the miR125b-binding site seed regions. Both wild-type and mutant 3′-UTRs were cloned downstream of the luciferase gene in the psiCHECK-2 luciferase vector. All of the above constructs were verified by sequencing.

Transfections were performed using Lipofectamine 2000 (Life Technologies) according to the manufacturer's instructions. 293T, 293T stably expressing DKK3 shRNA (DKK3 shRNA), or 293T-NC plasmid cells were transfected with the appropriate plasmids in 24-well plates. Cells were harvested and lysed for luciferase assays 48 h after transfection. The transfection experiments were performed in triplicate for each plasmid construct.

### Western blot

For western blotting, cells were lysed in lysis buffer containing 50 mM Tris-HCl, 150 mM NaCl, 0.5% NP-40, 1 mM Na_3_VO_4_, 10 mM NaF, 2 mM PMSF. Equal amounts of cell extracts were separated on SDS-PAGE gels, transferred onto nitrocellulose membranes, and incubated with the appropriate antibodies. The following antibodies were used: rabbit polyclonal IgG anti-DKK3 (Santa Cruz Biotechnology, Dallas, TX, USA), mouse monoclonal IgG anti-GATA4 (Santa Cruz Biotechnology), anti-tubulin (Santa Cruz Biotechnology), and anti-GAPDH (Sigma-Aldrich, St. Louis, MO, USA). Bands were scanned using a ChemiDoc XRSb Imaging System (Bio-Rad, Hercules, CA, USA). The absorbance of the bands was analyzed using image analysis software (Image J 1.48).

### Luciferase assay

For transfection of luciferase reporters, 293T cells or Huh6 cells grown in 24-well dishes were transfected with luciferase constructs, renilla plasmid, and/or miRNAs using Lipofectamine 2000 (Invitrogen). Transfected 293T cells in each well of the 24-well dishes were lysed with 100 μL of lysis buffer. 20 μL of the cell extract was used to measure luciferase and renilla activity on a GloMax® 20/20 Luminometer (Promega) using the Dual luciferase reporter assay system (Promega). Firefly luciferase activity was normalized to Renilla/luciferase as an internal control.

### Chromatin immunoprecipitation

A total of 5×10^7^ cells in the logarithmic growth phase were cross-linked with 1% formaldehyde. Then, 500 μL of cell lysis buffer was added and the cells were incubated on ice for 5 minutes. ChIP was conducted according to the manufacturer's instructions (26156, Thermo). Chromatin was sonicated into 200~1000 bp fragments and centrifuged at 12,000 rpm at 4°C for 15 minutes. The supernatant was collected and 20 μL samples of fragmented DNA were assigned to the experimental, control, and input groups. 5 μg of GATA4 rabbit mAb (5024s, Cell Signaling Technology) was added to the experimental group and 5 μg of normal rabbit immunoglobulin G (IgG) (2727, Cell Signaling Technology) was added to the control group, followed by incubation overnight at 4°C. Ten percent of chromatin prior to immunoprecipitation was used as the input control, and a nonspecific antibody (rabbit anti-IgG; BD Biosciences) was used as the negative control. The precipitated DNAs were subjected to PCR to amplify the GATA4-binding sites using primers specific for miR125b (forward, 5′-ACCTATCTCTGCTACTTATTTTATG-3′; Reverse, 5′-CTATTTATCAGCACAGTTACTGG-3′). PCR was performed as follows: 98°C for 5 min, 30 cycles of 98°C for 30 s, 55°C for 20 s, and 68°C for 20 s, and a final extension step at 68°C for 5 min. The amplified fragments were then resolved electrophoretically on a 1.2% (w/v) agarose gel and verified by DNA sequencing.

### Migration and invasion assays

To measure cell migration, transfected Huh6 cells were evenly seeded into 9 wells of 24-well dishes at 5×10^4^ cells/well and allowed to attach by culturing for 24 hr. Two wounds were then made in both vertical and horizontal directions across each well using a 10 μL pipette tip, and the cells were then cultured in fresh medium for additional 24 or 48 hr. At each time point, images along the longitude of the wounds were taken using a phase contrast microscope and wound areas were measured using NIH Image J software.

To measure cell invasion, the upper wells of the 24-well transwell chamber (BD Biosciences, Bedford, MA) were pre-coated with 0.05 mg/mL purified bovine collagen (Advanced Biomatrix, San Diego, CA) and then seeded with 2.5×10^4^ cells in serum free-medium. The upper wells were placed into lower wells containing regular culture medium, and cells were cultured for 24 hr. Subsequently, cells in the upper wells were removed using a cotton swab, the wells were washed three times with PBS, and cells in the lower wells were stained with 4% paraformaldehyde/0.1% Crystal violet (Sigma Aldrich, St. Louis, MO) for 10 min and air dried. The filters were removed from the wells and mounted on slides. Cells were counted on three replicate filters, and the average numbers of cells were used for analysis.

### Cell proliferation assays

Transfected Huh6 cells were evenly seeded into 9 wells of 24-well dishes at 1×10^4^ cells/well. After 24, 48, or 72 hrs of culture, cells from three wells were detached by trypsinization and re-suspended in 1 mL of culture medium. The total number of cells in each well was determined using a Coulter Counter (Beckman Coulter, Inc., Brea CA). The average number of cells in each well was used for evaluating increases in cell number at each time point.

### Tumor formation assay

For the *in vivo* tumor formation assays, 2×10^6^ Huh6 cells transfected with vectors or negative control were suspended in 200 μL of phosphate-buffered saline and injected into the tail vein of nude mice (15 in each group, female nu/nu, purchased from Shanghai Laboratory Animal Center of China). The mice were killed 4 weeks later, and the livers and tumor tissues derived from various organs were dissected and examined. The tumors were weighted and tumor sizes were monitored by measuring the length (L) and width (W) with calipers. Tumor volumes were calculated using the formula (L×W^2^)×0.5. All animal studies complied with the animal use guidelines of Fourth Military Medical University, and the protocols were approved by the ethics committee of Xiamen University.

### Statistical analysis

SPSS 12.0 software (SPSS, Chicago, IL, USA) was used for statistical analysis. Experimental data are expressed as the means ± S.E. Differences between means were assessed using Student's t-tests, ANOVAs, or X^2^-tests. Significant differences were determined by ANOVAs followed by *pos hoc* comparisons with Fisher's protected least significant difference test. Data were considered statistically significant at *P*<0.05.

## SUPPLEMENTARY MATERIALS FIGURES


