# Supplementary material for: GATA4 promotes hepatoblastoma cell proliferation by altering expression of miR125b and DKK3
Source: Oncotarget. 2016 Oct 24;7(47):77890–901. doi: 10.18632/oncotarget.12839 (PMC5363629; doi:10.18632/oncotarget.12839)
Supplement: Supplementary file 1 [file oncotarget-07-77890-s001.pdf]

GATA4 promotes hepatoblastoma cell proliferation by altering expression of miR125b and DKK3

SUPPLEMENTARY FIGURES

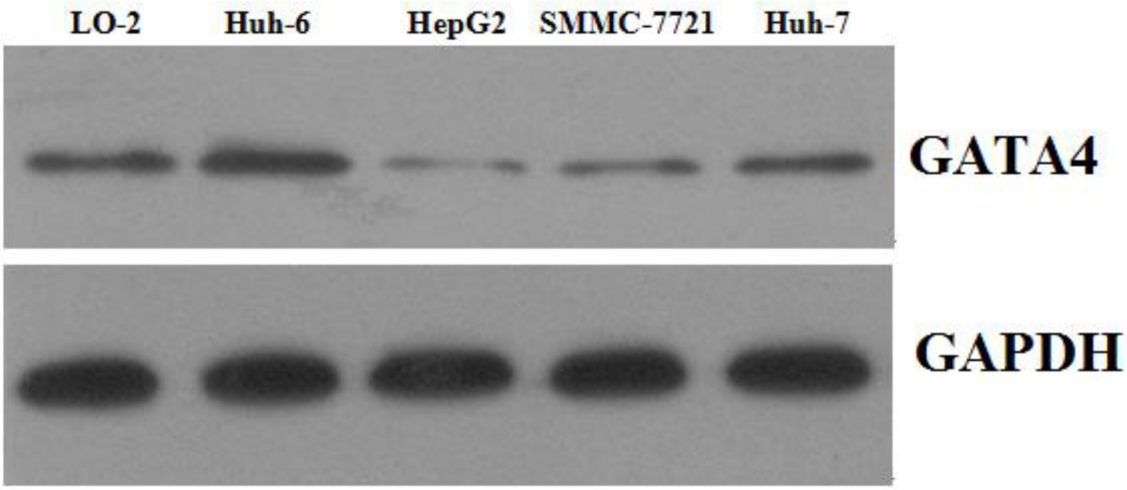

Supplementary Figure S1: Expression of GATA4 in five hepatic cell lines.

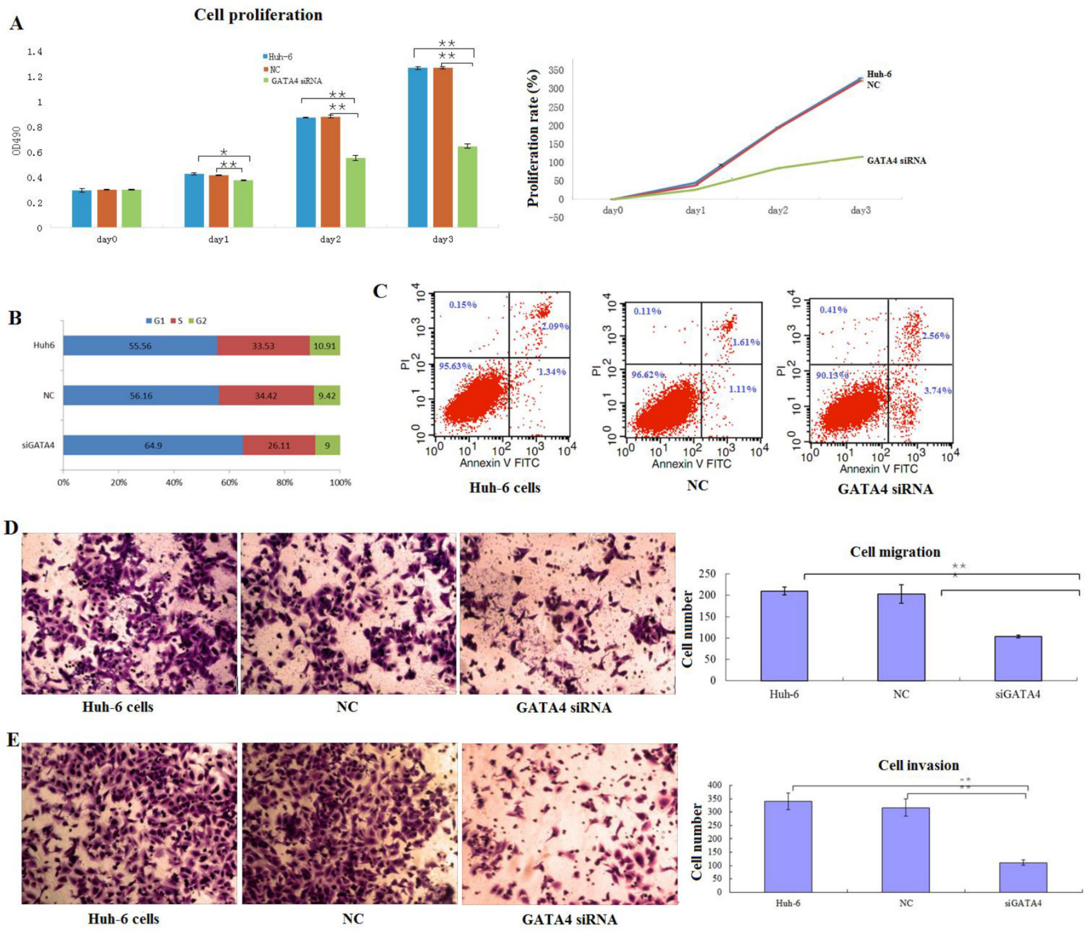

Supplementary Figure S2: Inhibition of GATA4 suppressed cell proliferation and survival *in vitro*. A. Proliferation curves of Huh6 cells after transfection of NC and GATA4 siRNA for 3 days. **B.** Analysis of cell cycles of Huh6 cells after transfection of NC and GATA4 siRNA. **C.** The apoptosis analysis of Huh6 cells, Huh6 cells treated with negative control shRNA (Huh6 NC) and Huh6 cells treated with GATA4 siRNA by using flow cytometry. The gated upper-right and lower-right regions represent apoptotic cells. **D.** Transwell migration assay using Huh6 cells transfected with NC or GATA4 siRNA (Left) and the migrated cell numbers in different groups (Right). **E.** Transwell invasion assay using Huh6 cells transfected with NC or GATA4 siRNA (Left) and the invaded cell numbers in different groups (Right). Error bards represent standard deviations from three repeated samples.

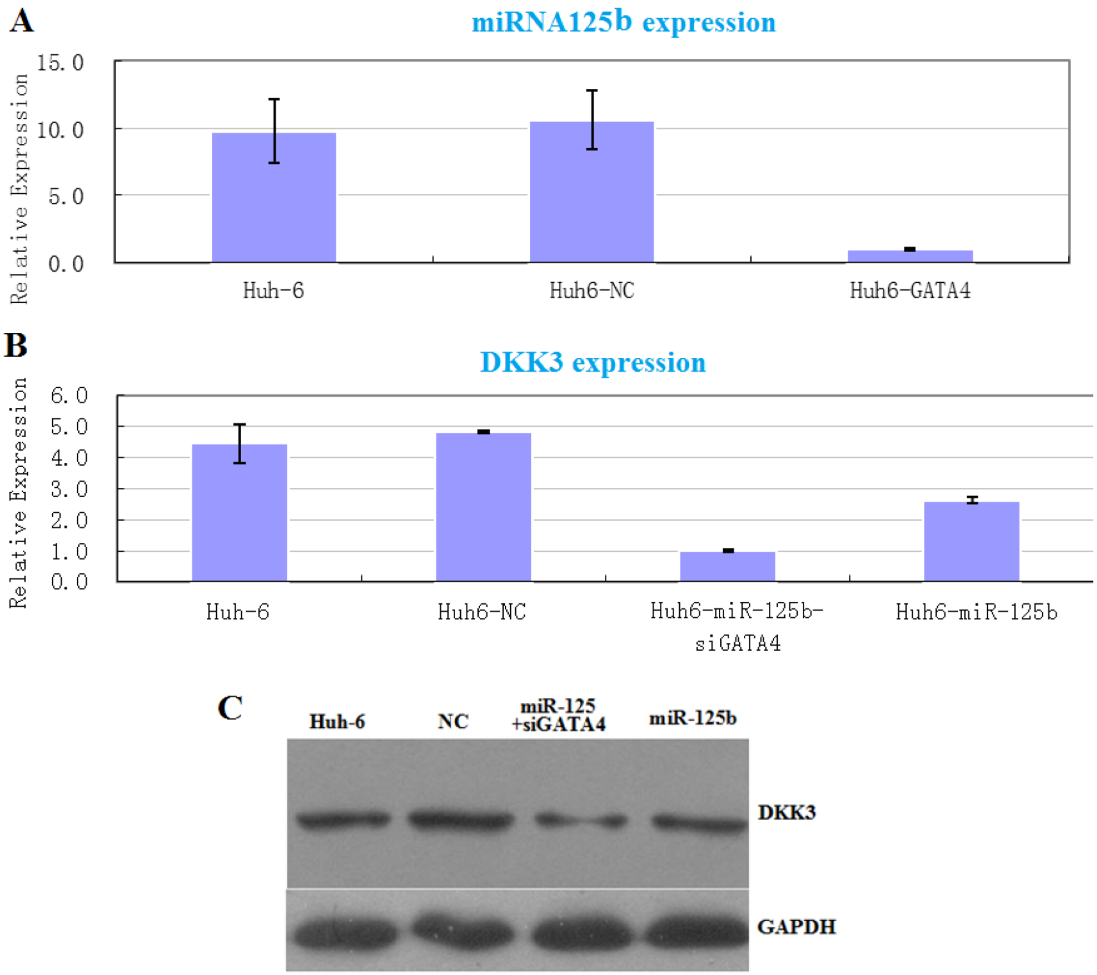

Supplementary Figure S3: GATA4 directly inhibited the expression of miR125b and miR125b directly inhibited the expression of DKK3. A. The expression of miR125b was examined in Huh6 cells, Huh6 transfected with empty vector (Huh6-NC) cells and GATA4-overexpressed Huh6 cells (Huh-GATA4) cells. The mRNA **B.** and protein **C.** expression of DKK3 in Huh6 cells, Huh6 transfected with empty vector (Huh6-NC) cells, Huh6 transfected with miR125b cells (Huh6-miR125b) and Huh6 co-transfected with miR125b and GATA4 siRNA cells (miR-125b-siGATA4). GAPDH as controls.
